# Podoplanin-expressing cancer-associated fibroblasts inhibit small cell lung cancer growth

**DOI:** 10.18632/oncotarget.3371

**Published:** 2015-03-24

**Authors:** Akiko Takahashi, Genichiro Ishii, Shinya Neri, Tatsuya Yoshida, Hiroko Hashimoto, Shigeki Suzuki, Shigeki Umemura, Shingo Matsumoto, Kiyotaka Yoh, Seiji Niho, Koichi Goto, Hironobu Ohmatsu, Kanji Nagai, Akihiko Gemma, Yuichiro Ohe, Atsushi Ochiai

**Affiliations:** ^1^ Division of Pathology, Research Center for Innovative Oncology, National Cancer Center Hospital East, Kashiwanoha, Kashiwa, Chiba 277-8577, Japan; ^2^ Division of Thoracic Oncology, National Cancer Center Hospital East, Kashiwanoha, Kashiwa, Chiba 277-8577, Japan; ^3^ Division of Thoracic Surgery, National Cancer Center Hospital East, Kashiwanoha, Kashiwa, Chiba 277-8577, Japan; ^4^ Department of Pulmonary Medicine and Oncology, Graduate School of Medicine, Nippon Medical School, Sendagi, Bunkyo City, Tokyo 113-0022, Japan; ^5^ Division of Thoracic Oncology, National Cancer Center Hospital, Tsukiji, Chuo City, Tokyo 104-0045, Japan

**Keywords:** podoplanin, small cell lung cancer, cancer-associated fibroblasts

## Abstract

Cancer-associated fibroblasts (CAFs) expressing podoplanin (PDPN) are a favorable prognosticator in surgically resected small cell lung cancer (SCLC). Here we explore whether CAFs expressing PDPN influence proliferation of SCLC cells. Compared with control group (SCLC cells co-cultured with CAFs-Ctrl), numbers of SCLC cells co-cultured with CAFs overexpressing PDPN were decreased. Suppression of PDPN expression by shRNA in CAFs resulted in increased numbers of SCLC cells. In surgically resected human SCLC specimens, the frequency of Geminin-positive cancer cells was significantly higher in the cases with PDPN-positive CAFs than in the cases with PDPN-negative CAFs. Thus CAFs expressing PDPN inhibit growth of SCLC cells, suggesting that CAFs expressing PDPN represent a tumor inhibitory stromal cell component in SCLC.

## INTRODUCTION

Cancer tissue is comprised of cancer cells, non-cancerous cells, and extracellular matrix (ECM), and these components constitute specific microenvironments. The tumor *microenvironment* is now recognized as a critical participant in tumor progression and drug resistance. Non-cancerous cell components include tumor-associated macrophages (TAMs), [1] immune cells, [2] and cancer-associated fibroblasts (CAFs). [2–4] Among the non-cancerous cell components, CAFs reside near the cancer nests and are the most abundant constituent cells of a tumor. [3] In many previous reports, CAFs have been shown to affect not only tumor cell growth and invasion, but also the host responses, such as angiogenesis and inflammation. [5–8] Therefore biological characteristics of CAFs play an improtant role in tumour progression and could be a prognostic indicator. [9] Recently, Cichon et al. reported that AKT2 phosphorylation in CAFs can induce epithelial cell invasion. [10] Guido et al. showed activation of the TGF-β pathway in CAFs induces their metabolic reprogramming and these metabolic alterations can spread among neighboring fibroblasts and greatly sustain the growth of breast cancer cells. [11] Therefore, CAFs can modify tumor metabolism.

Lung cancer is classified into two main subtypes: small cell lung carcinoma (SCLC) and non-small cell lung carcinoma (NSCLC). SCLC represents approximately 14%–20% of primary lung carcinomas [12–14] and has a more rapid doubling time; it also exhibits the earlier development of widespread metastases. Thus, this disease is highly aggressive, and approximately 60%–70% of patients have disseminated disease at the time of diagnosis. The current treatments for SCLC are radiation therapy, chemotherapy, or a combination of these treatments. However, a complete cure is presently difficult. Therefore, novel strategies are required for the treatment of SCLC.

Podoplanin (PDPN) is a 162-amino acid transmembrane sialoglycoprotein. [15–19] CAFs expressing PDPN have been confirmed in various tumors, and these cells have attracted great attention as a prognostic factor. [20, 21] We previously reported that PDPN-positive CAFs were found in some cases of lung cancer and that the presence of PDPN-positive CAFs predicted a poor outcome among patients with adenocarcinoma and squamous cell carcinoma. [22–24] In an animal model, we found that human fibroblasts overexpressing PDPN enhanced the tumor formation of human lung adenocarcinoma cell lines, and PDPN was a functional protein responsible for the promotion of tumor formation via enhanced RhoA activity in CAFs. [25, 26] On the other hand, we reported that SCLC patients with PDPN-positive CAFs who underwent surgery had a significantly better prognosis than those with PDPN-negative CAFs (overall survival: *P* < 0.05, relapse-free survival: *P* < 0.05, and 5-year overall survival: 74% vs. 46%). [27] So, we discovered that the presence of PDPN-positive CAFs in SCLC had a favorable prognostic value, unlike the situations for lung adenocarcinoma and squamous cell carcinoma. [27] Therefore, the extrinsic role of PDPN-positive CAFs in the SCLC progression process is likely to differ from that in adenocarcinoma and squamous cell carcinoma, with PDPN-positive CAFs possibly having a tumor suppressive effect in SCLC.

To test this hypothesis, we focused on the influence of CAFs expressing PDPN upon SCLC proliferation. In the present study, we examined the potency of CAFs expressing PDPN on the growth of SCLC using an *in vitro* co-culture model and surgically resected samples from humans.

## MATERIALS AND METHODS

### Cell cultures

Human small cell carcinoma cell lines (NCI-H69; ATCC#HTB-119 and NCI-H82; ATCC#HTB-171) and human adenocarcinoma cell lines (PC9; RIKEN BioResource Center#RCB4455) were originally purchased from ATCC or RIKEN BioResource Center and stocked at our institution. The CAFs were obtained from surgically resected small cell carcinoma specimens (Supplementary Table 1), and were cultured according to a previously described method. [28, 29] NCI-H69 and NCI-H82 were cultured in RPMI1640 (SIGMA-Aldrich, MO) containing 10% fetal bovine serum (FBS; Nichirei Bioscience, Japan) and 1% penicillin and streptomycin (SIGMA-Aldrich). The CAFs were cultured in MEM alpha (Life Technologies Corporation [Gibco], CA) supplemented with 10% FBS and 1% penicillin and streptomycin.

### Transfection

The lentiviruses were produced using 293T cells transfected with PCAG-HIV, CMV-VSV-G-RSV-Rev (RIKEN BioResource Center), and either PDPN-wild type (WT) vector (CSII-CMV-RfA-IRES2-Venus; RIKEN BioResource Center), CSII-CMV-mRFP1 (RIKEN BioResource Center), or PDPN short hairpin (sh) RNA vectors (CS-H1-shRNA-EG; RIKEN BioResource Center). [25, 26] Transfection was achieved using LipofectAMINE 2000 reagent (Invitrogen, CA) according to the manufacturer's instructions. The vector-containing medium was filtered through a 0.45 μm filter, and 8 μg/ml of Polybrene (SIGMA) was added for target cell transduction. The transduction efficiency was evaluated using a flow cytometry analysis to detect the positivity of mRFP-labeled cancer cells and Venus-labeled CAFs (Supplementary Figure 1). We used CAFs 1122 in the PDPN overexpression study and CAFs 1105 in the PDPN knockdown study (Supplementary Table 1).

### Co-culture methods

**Figure 1:**
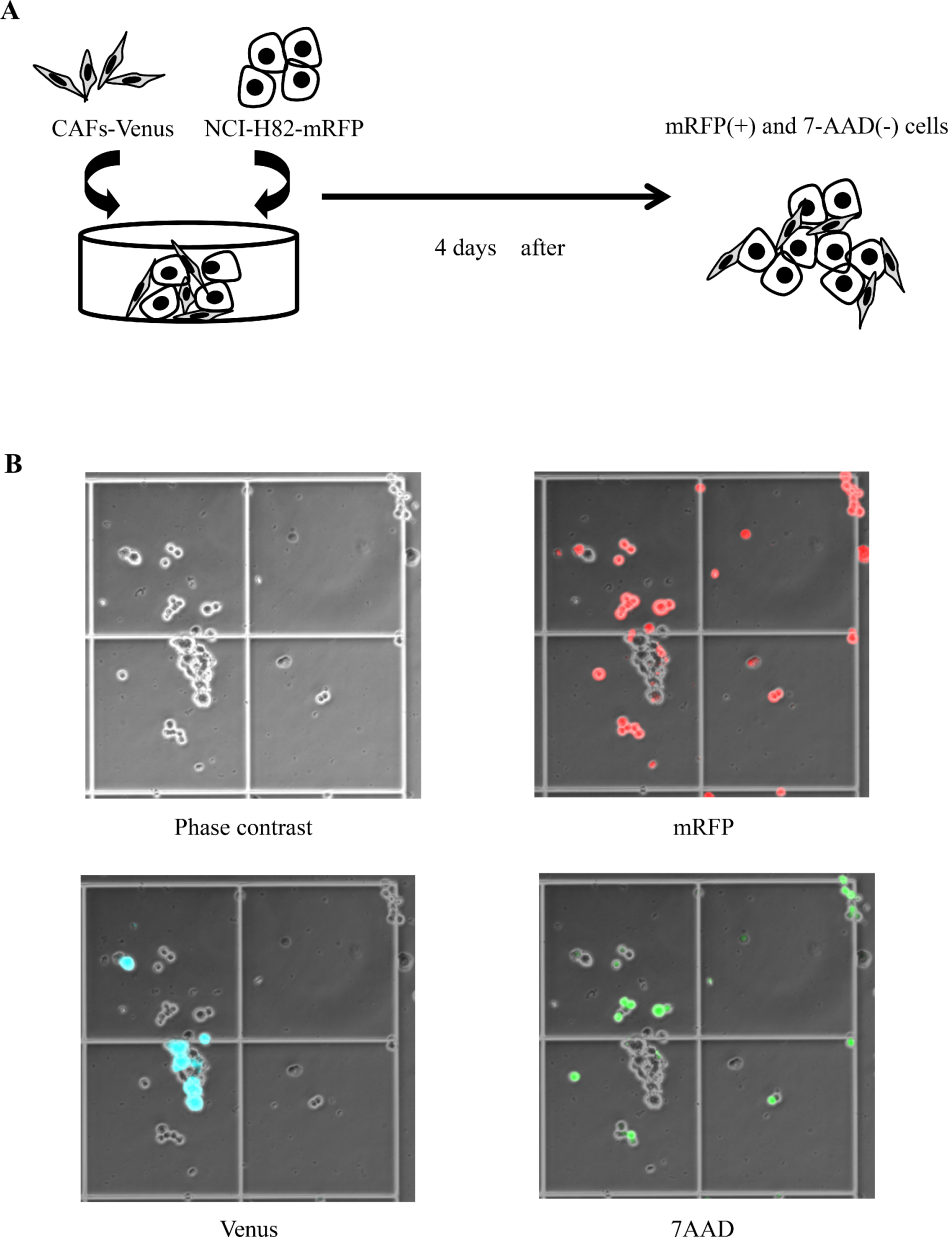
Co-culture model of SCLC cell lines and cancer-associated fibroblasts (CAFs) **(A)** Scheme of experimental procedure. **(B)** Phase contrast image (left upper), mRFP-labeled cancer cells (right upper), Venus-labeled CAFs (left lower), and 7AAD-positive cancer cells (right lower) as viewed using an optical microscope.

The mRFP-labeled cancer cells (2 × 10^5^ cells) in RPMI1640 and the Venus-labeled CAFs (2 × 10^5^ cells) in MEM alpha were equally mixed and plated onto a 60-mm dish (Figure 1A). On day 4, the number of mRFP-labeled cancer cells and the number of 7AAD (Beckman Coulter, CA)-positive cancer cells were measured using hemocytometers with an inverted-type fluorescence microscope (BZ-9000; KEYENCE, Japan) (Figure 1B). We defined dead tumor cells as both 7AAD-positive and mRFP-positive cells. On the hand, viable tumor cells were defined as mRFP-positive and 7AAD-negative cells. We calculated the percentage of cancer cells in each experiment using a denominator that represented the average value of the control group.

### Animal studies

NCI-H82 (1 × 10^6^ cells) and Venus-labeled CAFs (1 × 10^6^ cells) were injected under the dorsal subcutaneous tissue of SCID mice (7–10 weeks of age, CLEA, Japan). The tumor length, width, and height were measured every week. The tumor volume was calculated as the product of a scaling factor of 0.52 and the tumor length, width, and height. All the experimental SCID mice were handled in accordance with the institutional guidelines established using the Animal Care Committee of the National Cancer Center East Hospital.

### Study of surgery specimens

During the period from January 2004 to October 2011, a total of 36 consecutive patients with small cell lung cancer underwent surgical resection at our institution (Table 1). The tumors were staged according to the seventh edition of the tumor-node-metastasis classification developed by the International Union Against Cancer. All the surgical specimens were fixed with 10% formalin and were embedded in paraffin. For immunohistochemical staining, the tissue sections were stained overnight at 4°C using mouse anti-human Geminin antibody (Leica Microsystems, Novocastra, Germany) at a final dilution of 1: 200. As for Geminin-positive cells, 6 hot spots in PDPN-positive CAFs-infiltrated areas were selected, and the number of Geminin-positive cancer cells were counted under a light microscope at a x400 magnification (0.0625 mm^2^/field). Two observers (A.T. and G.I.) who were unaware of the clinical data independently reviewed all the pathological slides. The frequency of Geminin-positive cancer cells in each case was calculated as the average of the results from 6 areas. The status of PDPN expression in the CAFs was determined by reference to the results published in our previous reports. [27, 30] We also examined the frequency of Geminin-positive cancer cells of invasive lung adenocarcinoma with a tumor size of 2–3 cm in diameter (Cases with PDPN-positive CAFs vs Cases with PDPN-negative CAFs, *n* = 20, each).

### Statistical methods

Differences in categorical outcomes were evaluated using the chi-square test. The standard Student *t*-test was used to determine significant differences compared with the control group. All the *P* values were 2-sided, and the significance level was set at less than 0.05. Analyses were performed using the statistical software JMP 9 (SAS Institute, NC). This study was conducted as part of a National Cancer Center institutional review board-approved protocol.

## RESULTS

### Number of SCLC after co-culturing with CAFs-PDPN

**Figure 2:**
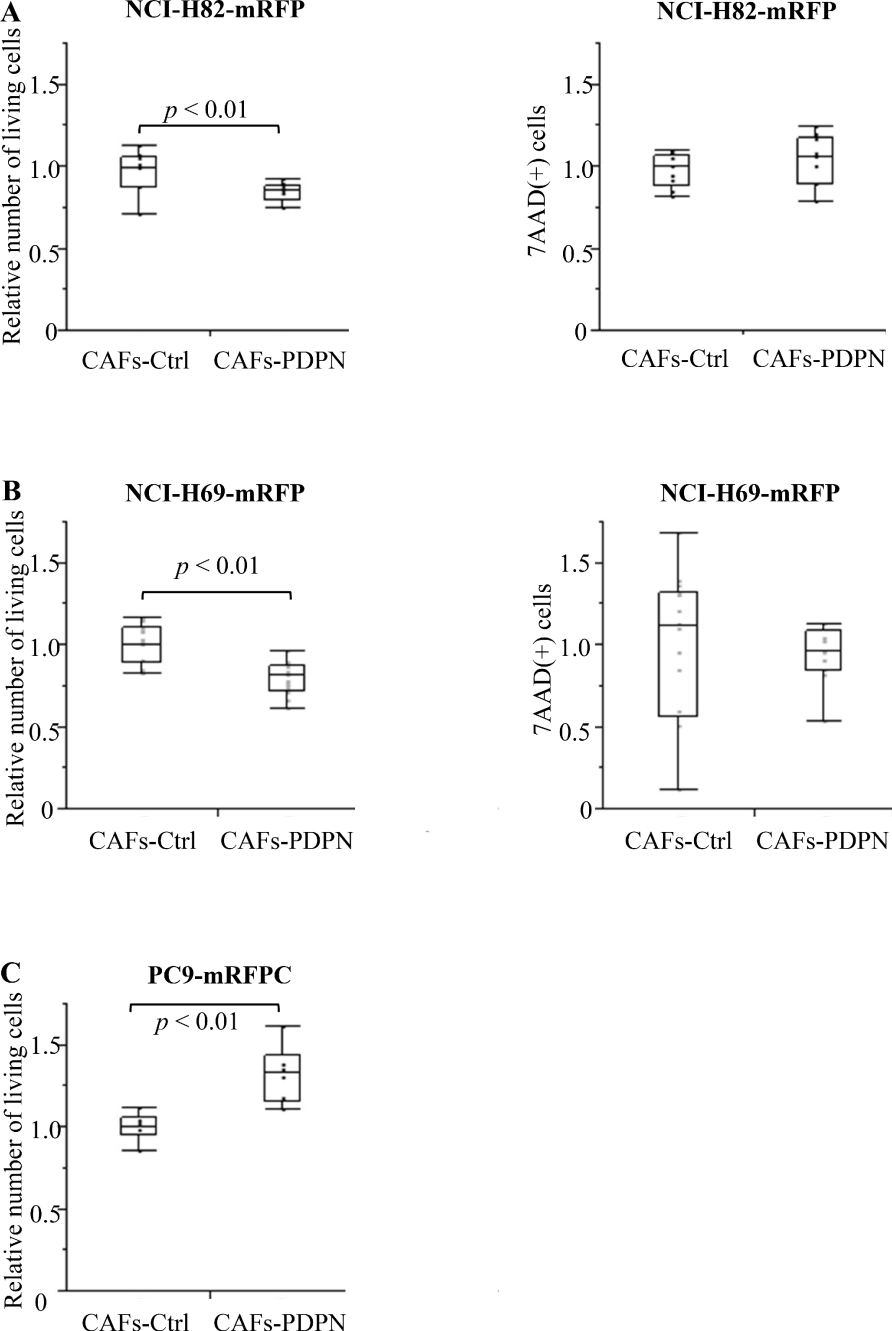
mRFP-labeled SCLC cell number after co-culturing with CAFs **(A)** Total viable NCI-H82 cell numbers after co-culturing with CAFs-Ctrl or CAFs-PDPN (left). Total 7-AAD-positive NCI-H82 cell number after co-culturing with CAFs-Ctrl or CAFs-PDPN (right). **(B)** Total viable NCI-H69 cell number after co-culturing with CAFs-Ctrl or CAFs-PDPN (left). Total 7-AAD-positive NCI-H69 cell number after co-culturing with CAFs-Ctrl or CAFs-PDPN (right). **(C)** Total viable PC9 cell number after co-culturing with CAFs-Ctrl or CAFs-PDPN.

We co-cultured mRFP-labeled SCLC cell lines (NCI-H82 and NCI-H69) with Venus-labeled CAFs expressing WT-PDPN (CAFs-PDPN) or CAFs-Control (CAFs-Ctrl); after 4 days, we then counted the number of viable NCI-H82 cells. When mRFP-labeled NCI-H82 was cultured alone, the number of 7-AAD-positive cells significantly decreased and the viable cell number increased to 127.6% of cancer cell co-cultured with CAFs-Ctrl (Supplementary Figure 2). Co-culture with CAFs-PDPN suppressed the viable NCI-H82 cancer cell number to 84.5% of the number of cancer cells after co-culturing with CAFs-Ctrl (*P* < 0.01) (Figure 2A). CAFs-PDPN did not affect the number of dead cancer cells (7-AAD-positive cancer cells, *P* = 0.59). We also used another SCLC cell line, NCI-H69, and confirmed similar results. Co-culture with CAFs-PDPN decreased the viable NCI-H69 cell number to 79.5% of the number of cancer cells after co-culturing with CAFs-Ctrl (*P* < 0.01) (Figure 2B).

In contrast to the SCLC cell lines, CAFs-PDPN increased the number of cancer cells belonging to the lung adenocarcinoma cell line PC9, compared with the number after co-culturing with CAFs-Ctrl (132.3%, *P* < 0.01) (Figure 2C).

### Number of SCLC after co-culturing with CAFs-shPDPN

**Figure 3:**
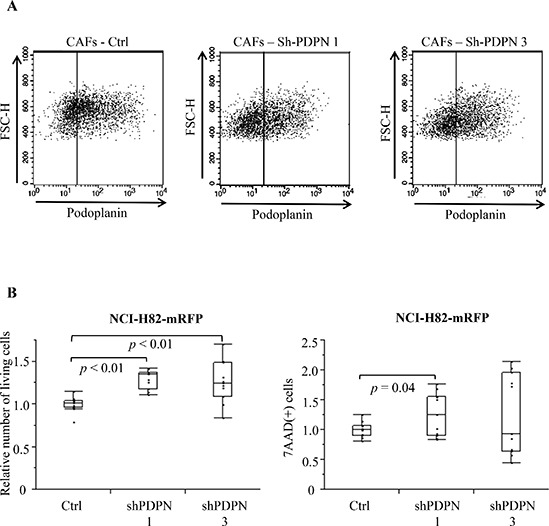
mRFP-labeled SCLC cell number after co-culturing with CAFs inducing shRNA PDPN **(A)** Flow cytometry analysis of PDPN expression in CAFs-Ctrl, CAFs-shPDPN1, and CAFs-shPDPN3. **(B)** Total viable NCI-H82 cell number after co-culturing with CAFs-Ctrl, CAFs-shPDPN1, or CAFs-shPDPN3 (left). Total 7-AAD-positive NCI-H82 cell number after co-culturing with CAFs-Ctrl, CAFs-shPDPN1, or CAFs-shPDPN3 (right).

We prepared two different PDPN knockdown CAFs (CAFs-shPDPN1 and CAFs-shPDPN3) [25] (Figure 3A). Co-culture with CAFs-shPDPN1 and CAFs-shPDPN3 significantly increased the number of viable NCI-H82 cancer cells to 129.2% and 125.6%, respectively, of the number of cancer cells after co-culturing with CAFs-Ctrl (*P* < 0.01 and *P* < 0.01). CAFs-shPDPN1 increased the number of dead cancer cells (*P* = 0.04), but co-culturing with CAFs-shPDPN3 did not have any effect (*P* = 0.25) (Figure 3B).

### Influence of CAFs-PDPN on the engraftment of NCI-H82 in mouse subcutaneous tissue

**Figure 4:**
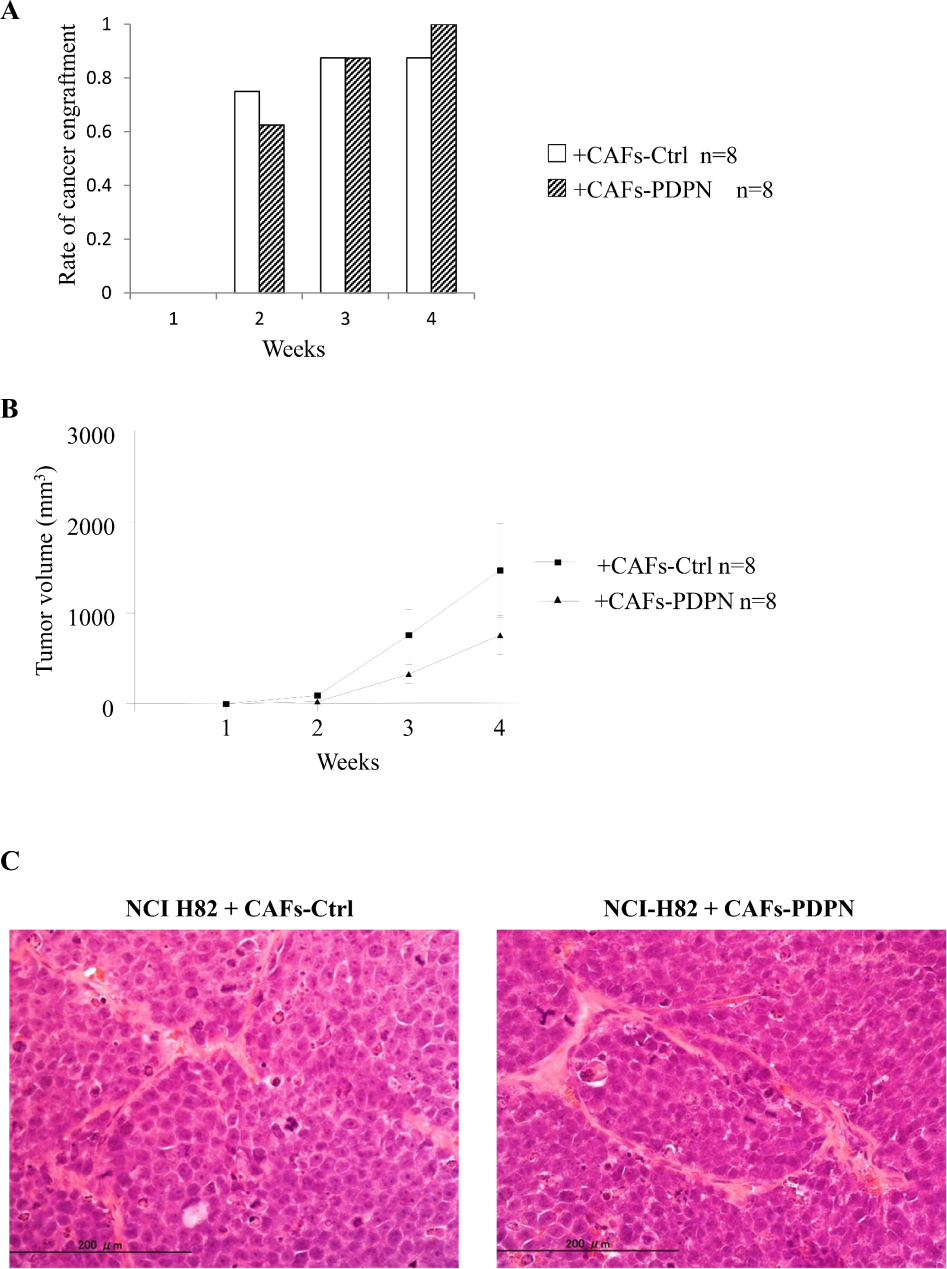
Engraftment rate and tumor volume of NCI-H82 injected with CAFs in mouse subcutaneous tissue **(A)** Rate of cancer engraftment at 1, 2, 3, and 4 weeks. **(B)** Tumor volume of engrafted tumors at 1, 2, 3, and 4 weeks. **(C)** Histological features of engrafted tumors at 4 weeks after tumor cell injection (H.E. staining) (left, group of CAFs-Ctrl; right, group of CAFs-PDPN).

Next, we examined whether CAFs-PDPN influences tumor engraftment or tumor volume. NCI-H82 cells were subcutaneously co-injected with either CAFs-PDPN or CAFs-Ctrl into SCID mice. The tumor formation rates of NCI-H82 cells co-injected with CAFs-Ctrl and of cells co-injected with CAFs-PDPN were 75% and 63%, respectively at 2 weeks and 88% and 100%, respectively, at 4 weeks (*P* = 0.59) (Figure 4A). The tumor volume resulting from co-injection with CAFs-Ctrl was larger than the tumor volume resulting from co-injection with CAFs-PDPN at 4 weeks (mean tumor volume: CAFs-PDPN, 757.1 mm^3^ vs. CAFs-Ctrl, 1469 mm^3^); however, the difference was not significant (Figure 4B).

Histological examination of tissue samples from NCIH-82 with CAFs-PDPN or CAFs-Ctrl revealed no difference in morphology, including both the tumor cell and the stromal cell components and the invasiveness into the surrounding tissues (Figure 4C).

### Frequency of Geminin-positive cancer cells in surgically resected SCLC with PDPN-positive CAFs cases

**Figure 5:**
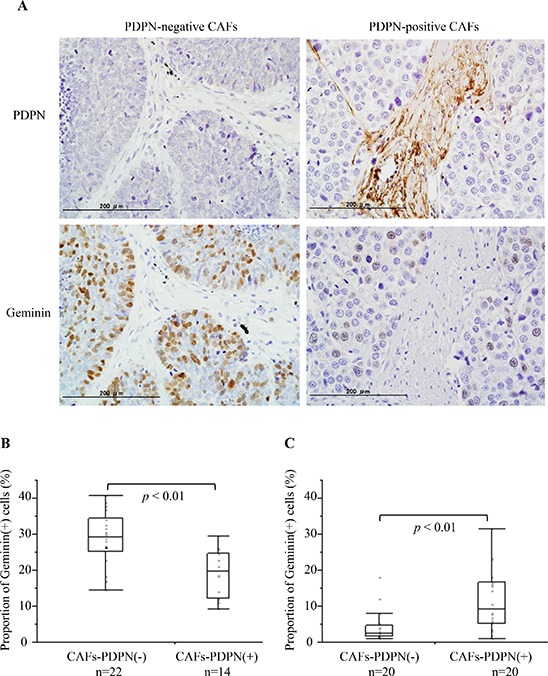
Frequency of Geminin-positive SCLC cells in surgical specimens with CAFs-PDPN and those without CAFs-PDPN **(A)** Immunohistochemical staining for PDPN (upper) and Geminin (lower). The left figure shows a case without CAFs-PDPN, while the right shows a case with CAFs-PDPN. **(B)** Proportion of Geminin-positive SCLC cells. **(C)** Proportion of Geminin-positive adenocarcinoma cells.

To verify the *in vitro* results, we examined the frequency of Geminin-positive cancer cells in cases with PDPN-positive CAFs and those with PDPN-negative CAFs among surgically resected SCLC specimens (Table 1). Fourteen of the 36 specimens (38.9%) were PDPN-positive CAFs cases. Among the cases with PDPN-positive CAFs, 19.0% ± 1.7% of the SCLC cells showed a positive reaction for Geminin. However, 28.8% ± 1.6% of the SCLC cells showed a positive reaction for Geminin among the cases with PDPN-negative CAFs (*P* < 0.01) (Figure 5A and 5B). We also examined the frequency of Geminin-positive cancer cells of invasive lung adenocarcinoma with a tumor size of 2–3 cm in diameter (Cases with PDPN-positive CAFs vs Cases with PDPN-negative CAFs, *n* = 20, each). Adenocarcinoma cells in the cases with PDPN-positive cases showed significant higher positive ratio for Geminin than in the cases with PDPN-negative CAFs (9.2% vs 2.5%, *p* < 0.01) (Figure 5C).

**Table 1:** Clinicopathological characteristics of the patients

Characteristics		***n*** = 36
Age (year)	< 70	18
	≥ 70	18
Gender	Female	4
	Male	32
Brinkman Smoking Index	< 1000	19
	≥ 1000	17
pT	T1a-T1b	23
	T2a-T4	13
pN	pN0	28
	pN1–2	8
Pathological stage	IA-IB	25
	IIA-IV	11
Vascular invasion	Absent	7
	Present	29
Lymphatic invasion	Absent	31
	Present	5
Pleural invasion	Absent	27
	Present	9
PDPN-positive CAFs	Absent	14
	Present	22

## DISCUSSION

Many previous reports have presented the tumor-promoting effects of CAFs on cancer cell growth, survival, and invasion. [2, 3, 7] However, CAFs consist of heterogeneous populations that have distinct phenotypes and functions; [3, 31] thus, the analysis of CAF subpopulations is needed. We have reported that the presence of CAFs expressing PDPN is a predictor of a poor prognosis among patients with lung adenocarcinoma and squamous cell carcinoma of the lung, [22–25] and PDPN itself has been shown to exert tumor-promoting effects using *in vitro* and *in vivo* models. Therefore, we considered CAFs expressing PDPN to represent a tumor-promoting component in adenocarcinoma and squamous cell carcinoma. In contrast to our expectations, however, the results obtained using surgically resected specimens of lung SCLC were opposite to the previous findings. [27] Based on these results, the present study was performed to evaluate the biological roles of CAFs expressing PDPN in SCLC for the first time.

The current study showed that CAFs-PDPN suppressed the growth of SCLC using an *in vitro* co-culture model and surgically resected SCLC specimens. Furthermore, PDPN was found to act as a functional molecule that influences the proliferation potency of SCLC based on experimental results obtained using PDPN-knockdown CAFs. We further examined whether recombinant human PDPN has the same effects on SCLC growth. rPDPN or rGFP as control recombinant protein were added to NCI-H82 cells, and the number of viable NCI-H82 cells was counted. As shown in Supplementary Figure 3A, 100 ng/ml of rPDPN suppressed the number of viable NCI-H82 cancer cells to 82.4%, compared with the number of cells after treatment with 100 ng/ml of rGFP (*P* < 0.01). On the hand, 100 ng/ml of rPDPN did not influence the number of viable PC9 cancer cells (Supplementary Figure 3B). Moreover the addition of rPDPN increased the CDT1 expression level (G1 associated protein) on day 1 as compared with control group. The addition of rPDPN decreased H3S10p expression level (G2 and M phase associated protein) on day 2 (Supplementary Figure 3C). These findings suggested the possibility that an extracellular region of PDPN might interact with SCLC as a ligand, the downstream signals of which might inhibit growth. The known receptor for PDPN is C-type lectin-like receptor 2 (CLEC-2), which is reportedly expressed by platelets, neutrophils, and dendritic cells. [17, 32–35] We examined the mRNA expression status of CLEC2; however, the SCLC cell lines that we used did not express a significant amount of CLEC2 mRNA (Supplementary Figure 4). Therefore, we think that signal transduction through a ligand (other than CLEC2) for podoplanin may have suppressed the growth of the SCLC cells after it came in direct contact with CAFs-PDPN. Further studies examining ligands for PDPN and the subsequent signaling pathways are needed for the clarification of CAFs-PDPN mediated inhibition of SCLC.

In our previous reports, CAFs-PDPN increased the subcutaneous tumor formation ratio of the lung adenocarcinoma cell line A549 when evaluated using SCID mice. [25, 26] However, the current study revealed that CAFs-PDPN did not influence either the tumor formation ratio or the tumor growth ratio (Figure 5). Moreover, they did not alter the histological growth pattern of SCLC. Considering these results, signaling via PDPN might not affect the factors involved in tumor formation in SCLC. The present *in vivo* results showing no significant differences in tumor growth can be explained as follows. First, a relatively small number of CAFs had survived and continued to exist in the subcutaneous tumor tissue at 3 to 4 weeks after injection with an SCLC cell line into SCID mice. Actually, we performed immunohistochemical staining of the engrafted tumors using anti-Venus antibody and confirmed that only a small number of Venus-positive fibroblasts were scattered within the engrafted tumors (data not shown). Therefore, the suppressive effect on the growth of SCLC exerted by CAFs-PDPN might be insufficient using this *in vivo* model. Alternatively, several kinds of stromal cells from the host mouse might have influenced the proliferation potency of SCLC.

The current result showed that CAFs-Ctrl inhibited the viable cell number of SCLC cells (Supplementary Figure 2), which was associated with higher cell number of 7AAD positive cells. To solve the molecular mechanisms how CAFs induced cell death of SCLC cells would be important for better understanding of cancer microenvironment created by SCLC cells and CAFs.

In conclusion, this study clearly showed that CAFs-expressing PDPN had an inhibitory effect on the proliferation of SCLC. In lung adenocarcinoma and squamous cell carcinoma, we considered CAFs-expressing PDPN to be a stromal cell component with a tumor-promoting phenotype. In SCLC, however, this type of CAFs might have a tumor inhibitory effect. Therefore, the identification of signal transduction via PDPN will become increasingly important for clarification of microenvironments consisting of SCLC cells and CAFs-PDPN. With the further elucidation of molecular mechanisms of how CAFs-PDPN influence the proliferative capacity of SCLC cells, novel method that directly target CAFs-PDPN or inhibit the PDPN-mediated signaling pathway might be one of the candidates for the treatment of SCLC.

## SUPPLEMENTARY FIGURES AND TABLES


