# Supplementary material for: Podoplanin-expressing cancer-associated fibroblasts inhibit small cell lung cancer growth
Source: Oncotarget. 2015 Mar 24;6(11):9531–41. doi: 10.18632/oncotarget.3371 (PMC4496236; doi:10.18632/oncotarget.3371)
Supplement: Supplementary file 1 [file oncotarget-06-9531-s001.pdf]

SUPPLEMENTARY FIGURES AND TABLES

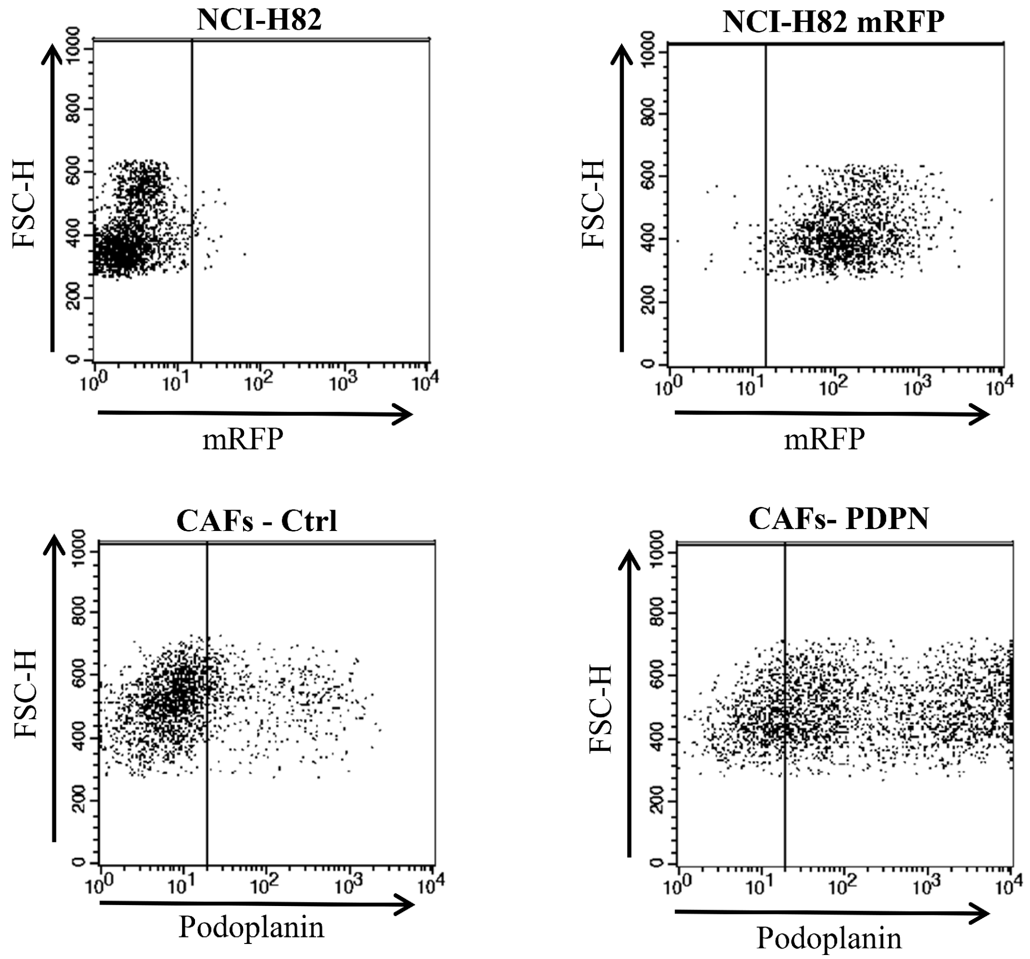

Supplementary Figure S1: Flow cytometry analysis of mRFP-labeled SCLC and Venus-labeled CAFs used in this study. Left upper, original NCI-H82, right upper, mRFP-labeled NCI-H82; left lower, PDPN expression of CAFs-Ctrl; and right lower, PDPN expression of CAFs-PDPN.

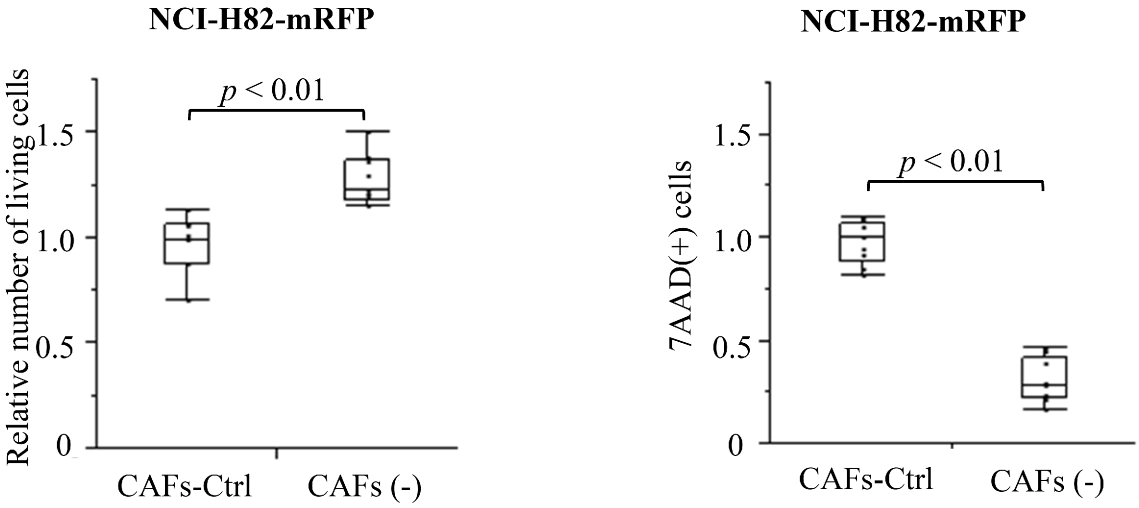

Supplementary Figure S1: mRFP-labeled SCLC cell number with and without CAFs. Total viable NCI-H82 cell number with and without CAFs-Ctrl (left). Total 7-AAD-positive NCI-H82 cell number with and without CAFs-Ctrl (right).

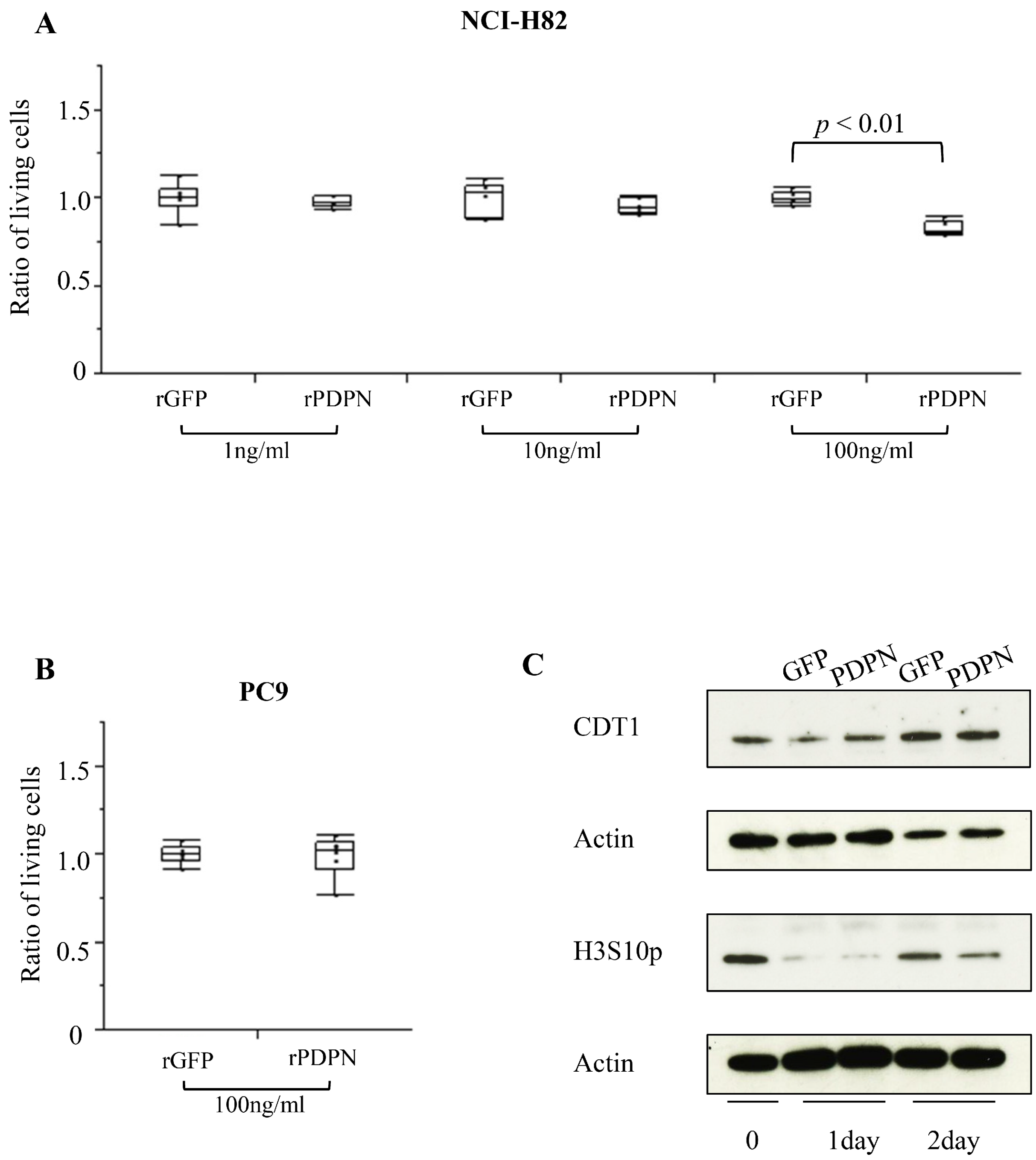

Supplementary Figure S1: NCI-H82 and PC9 cell number after treatment with recombinant PDPN. **(A)** Total viable NCI-H82 cell number after co-culturing with rGFP or rPDPN (left, 1 ng/ml of recombinant protein; middle, 10 ng/ml of recombinant protein; and right, 100 ng/ml of recombinant protein). **(B)** Total viable PC9 cell number after co-culturing with 100 ng/ml of rGFP and rPDPN. rPDPN, a single and non-glycosylated polypeptide chain (99–207 a.a.) (ProSpec Tany TechnoGene Ltd., Israel; rGFP, Life Technologies Corporation). **(C)** Analysis of CDT1 and H3S10p expression of NCI-H82 cells on 0, 1, and 2day treatment using anti-CDT1 Ab (Cell Signaling Technology, MA) and anti H3S10p Ab (Abcam, UK).

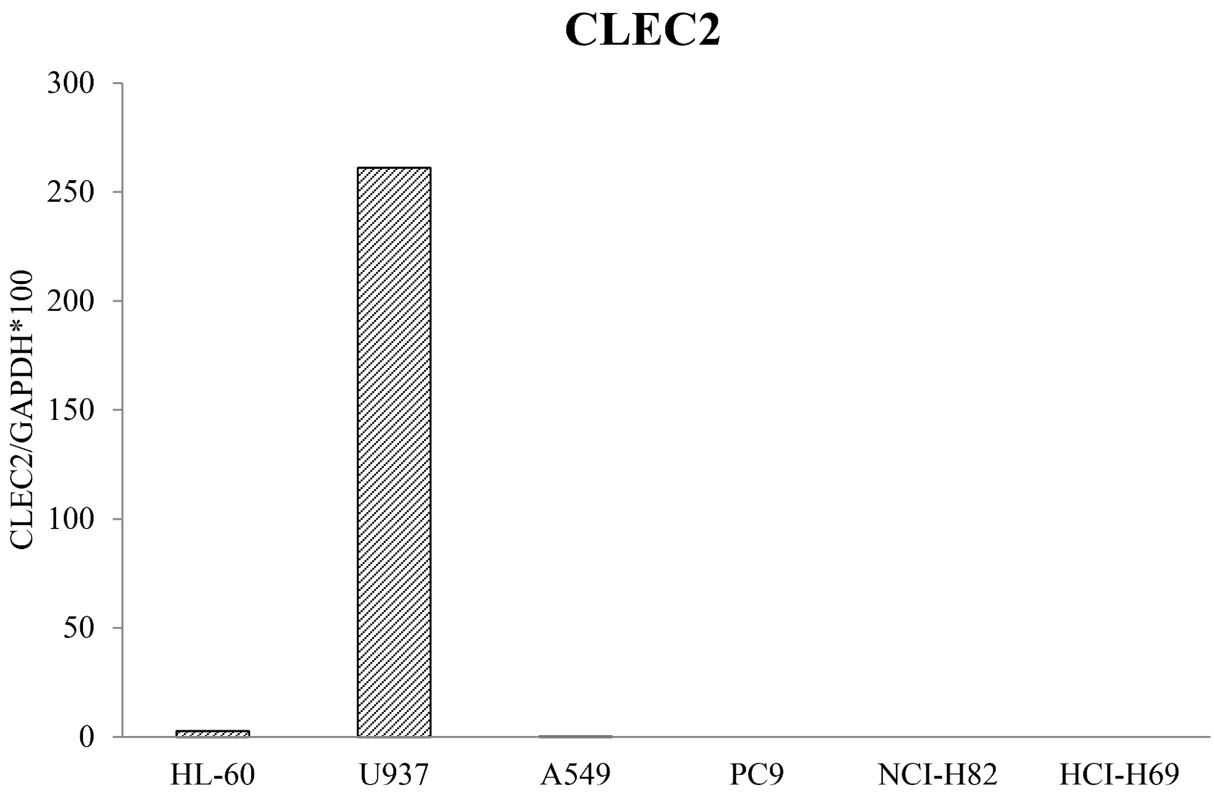

Supplementary Figure S1: RT-PCR analysis of CLEC2 mRNA in tumor cell lines. The mRNA level of CLEC2 was analyzed in 6 human cancer cell lines. The U937 cell line, which is a human monohistiocytic leukemia cell line, was used as a positive control for CLEC2.

Supplementary Table S1: Information of cancer-associated fibroblasts

CAFs	Age	Sex	Pathological diagnosis
CAF1105	73	Male	SCLC<br>pT2bN0M0, stage IIA
CAF1122	74	Male	SCLC<br>pT2bN1M0, stage IIB
